# Optotransduction Pathway, Exploring Connections with Inflammation

**DOI:** 10.3390/biom16060859

**Published:** 2026-06-11

**Authors:** Alessandro Ravoni, Veronica Paparozzi, Tiziana Guarnieri, Cecilia Sanzini, Luigi Manni, Christine Nardini

**Affiliations:** 1 Consiglio Nazionale delle Ricerche, Istituto per le Applicazioni del Calcolo “Mauro Picone”, 00185 Roma, Italy; alessandroravoni@cnr.it (A.R.); veronicapaparozzi@cnr.it (V.P.); tiziana.guarnieri@unibo.it (T.G.); ceciliasanzini@gmail.com (C.S.); 2 Dipartimento di Scienze Biologiche, Geologiche e Ambientali (BIGEA), University of Bologna, 40100 Bologna, Italy; 3 Centro Interdipartimentale di Ricerca Industriale—CIRI Scienze della Vita e Tecnologie per la Salute, Università di Bologna, 40126 Bologna, Italy; 4 Consiglio Nazionale delle Ricerche, Istituto di Farmacologia Traslazionale, 00185 Roma, Italy; luigi.manni@ift.cnr.it

**Keywords:** optotransduction, photobiomodulation, inflammation, systems biology markup language, pathway, network biology

## Abstract

The ability of cells to translate optical radiation into biochemical signals, i.e., optotransduction, plays an important role in the life sciences, including the development of emerging therapeutic strategies, with a relevant influence on inflammation. However, a systemic understanding of the molecular pathways underlying the transduction of these physical stimuli is still lacking. In this work, we present a molecular map of optotransduction reconstructed from the literature and provide its representation as pathway, using the standard Systems Biology Markup Language. This representation enables network-based analyses and allows us to explore the differential effect of stimuli wavelengths and, for the first time, the systematic overlap with other forms of physical transduction, namely mechanotransduction.

## 1. Introduction

Signal transduction represents the fundamental process by which physical stimuli are converted into biochemical signals, enabling the transmission of information both within and between cells. In a cell, these stimuli interact with specific components, triggering responses or cascades of biochemical reactions essential for homeostasis and functional adaptation. In this work, we study the molecular mechanisms downstream of the conversion of optical radiation beyond the specialized processes occurring in the eye. In the following, to emphasize the concept of transduction, while focusing on optical radiation, we refer to the process as optotransduction, distinct from mechanotransduction and other types of physical transduction [1].

It was long believed that optical stimuli could exclusively influence specialized cells in the retina; more recent studies, however, have shown that skin cells, such as fibroblasts, keratinocytes and melanocytes, can detect sunlight through chromophores and a series of light-sensitive proteins called opsins [2,3,4]. Activation of these receptors can initiate a variety of intracellular signaling cascades that regulate multiple biological processes, including cell proliferation, pigmentation, stress responses and the modulation of inflammatory pathways [1,4,5]. Optotransduction, therefore, represents a key mechanism through which optical stimuli can be translated into functional changes in a systemic and impactful way, given the ubiquity of fibroblasts and the extension of skin.

In the clinical landscape, the ability of tissues to respond to optical stimuli underlies several therapeutic approaches (in dermatology, neurology and regenerative medicine) based on controlled light exposure, including Photobiomodulation (PBM), Low-Level Laser Therapy (LLLT) [5,6,7,8,9,10] and, more recently, experimental greenlight exposure in prodromal Alzheimer patients [11], to name a few. These techniques involve the application of specific wavelengths, typically ranging from 400 to 1200 nm, delivered at low power densities to induce photobiological effects without causing thermal damage or cytotoxicity. Among the most interesting outcomes is the potential anti-inflammatory effect associated with these therapies. Several studies have shown that tissues exposed to low-intensity light exhibit a local reduction in edema, oxidative stress markers and pro-inflammatory cytokines [6]. Despite these clinical benefits, to our knowledge, the cellular signaling pathways responsible for these actions are not fully understood, in particular due to jeopardized information, lacking organization into a coherent systemic framework.

In this context, constructing a comprehensive representation of the molecular actors involved in optotransduction becomes particularly useful. In the present work, we integrated information scattered across the literature to build a systematic representation of the interactions between optical stimuli and inflammatory processes.

The identified molecular interactions have been organized within a network-based formalism, a mathematical approach at the basis of the well assessed Systems [12] and Network Biology [13] research areas. Systems and Network Biology in fact, enable capturing the complexity of biological systems by representing the multiple interactions occurring among the molecular entities involved. Networks or maps or graphs (G), can be formally represented by three sets, namely nodes (N, representing the molecular entities), edges (E, representing the connections among molecular entities, like biochemical reactions) and attributes (A, that further qualify the nature of nodes and edges), summarized as G = {N,E,A}. We adopt here the Systems Biology Markup Language (SBML, [14]) to describe the graph G in human- and machine-readable format to enable manual inspection, as well as automated computational analyses. We also exploit the associated System Biology Graphical Notation (SBGN, [15]) for visual inspection using standard notations, as discussed in Methods. This globally ensures uniformity, reliability, and reproducibility of the representation.

Among the computational analyses enabled by this representation, we perform *diffusion* analysis [16] (see Section 2.3 for further details) to explore the differential biological processes elicited by distinct optical stimuli, i.e., distinct wavelengths. Finally, the map allows for the identification of overlap with the pathway of mechanotransduction recently updated by our group [17]. Given the very limited representation of molecular physical transductions, this represents a unique opportunity to explore commonalities (see Ref. [1] for a review of known shared transduction mechanisms and their therapeutic applications).

This work highlights the potential of standardized (SBML, SBGN) biological maps (aka in silico pathways) and supports the broader perspective of progressively integrating different representations of stimuli transduction mechanisms within a unified framework. The first tangible result is the construction of a map collecting in one place the molecular actors involved in optotransduction. The second result represents a more remarkable première, as it identifies the potential equivalence of stimuli of a completely different nature (optical and mechanic). Thanks to the identification of the overlap between opto- and mechanotransduction, we can highlight a specific shared mechanism, experimentally observed under optical and mechanical experiment, but never before identified as common, with this level of detail. Finally, our work presents the differential effects elicited by optical stimuli, offering a rationale to support the emerging empirical optotransduction-based therapies. It is to be remarked that these computational results represent a valuable starting point for cost-effective experimental validation. This is indeed the main role and power of computational biology.

To summarize the rationale of the present study, the workflow consists of the following steps: (i) collection of molecular interactions from the literature and construction of the network in SBML format for computational analysis and in SBGN format for visual inspection; (ii) diffusion-based analysis followed by enrichment analysis to identify the biological processes elicited by distinct optical stimuli; and (iii) overlap analysis between optotransduction and mechanotransduction to identify and discuss shared molecular pathways between the two transduction processes, with particular focus on inflammation-related mechanisms.

This work also sets the bases for future exploitations (beyond the potential of this founding map) that identify *emergent properties*, i.e., characteristics that become observable only when a system is studied in its complexity. These emergent properties could be of translational interest when integrating the optotransduction map with other relevant pathways, such as drugs metabolism or diseases of interest, highlighting functional relations that might remain hidden when analyzing isolated aspects of optotransduction [18].

In the following we will describe the methods and results concerned with: (i) the literature-based construction of the optotransduction map following the SBML/SBGN standards; (ii) the overlap between optotransduction and mechanotransduction using two different computational approaches; (iii) the simulated effect of stimuli of different wavelength in terms of the biological function they can elicit. We enforce once more that, although extremely valuable in terms of robustness and cost-effectiveness, these results, like all computation simulations, represent well-thought hypotheses whose final validation cannot preclude from wet lab/clinical experiments.

## 2. Materials and Methods

### 2.1. Design of the Optotransduction Pathway

We collected the relevant literature [3,4,5,6,7,8,9,19,20,21,22,23,24,25,26,27,28,29,30,31,32,33,34,35,36,37,38,39,40,41,42,43,44,45] from PubMED (https://pubmed.ncbi.nlm.nih.gov/) and Google Scholar (https://scholar.google.com/) using the keywords, PBM, LLLT, Opsin, Phototransduction, Anti-inflammatory effects, Inflammation, and adding specific information on the Aryl Hydrocarbon Receptor, for its role as a mediator of environmental signals and inflammation [17].

All molecular entities and reactions mentioned in the literature were manually organized into the SBML Level 2 Version 4 format [14], a well-defined standard language used to represent in silico pathways in a human- and machine-readable format. This enables structured models’ descriptions by organizing biochemical actors (species) and the processing governing their interactions (reactions) into eXtensible Markup Language (XML) data structures. The core components common to all SBML Levels and Versions consist of Compartment, Species and Reaction objects, hereafter referred to as entities in a broad sense. Each entity is defined by a set of mandatory and optional attributes (e.g., “metaid”, “id”, “name”) that among other functions ensure internal model referencing, as well as additional information (including relevant publications) incorporated through the Notes and Annotation tags. Detailed specifications are provided in the Supplementary Materials (Section Optotransduction Map). Notably, the Annotation element was employed to ensure standardized entities’ identification in accordance with the MIRIAM [46] guidelines, by linking each entity to the appropriate external database resources. Reference databases for species were selected based on their biochemical nature: the Universal Protein Knowledgebase (Uniprot, [47]) for proteins, National Center for Biotechnology Information Gene (NCBI, [48]) for genes, Chemical Entities of Biological Interest (ChEBI, [49]) for small molecules, Systems Biology Ontology (SBO, [50]) for stimuli, and Gene Ontology (GO, [51]) Cellular Components sub ontology for cellular compartments.

To match these definitions to the general graph notation introduced in Section 1, an SBML model can be represented as a graph G = {N,E,A} as follows: species correspond to the nodes N; reactions correspond to the edges E, where each edge may connect multiple nodes at either end (i.e., hyperedges) or link nodes (i.e., *modifier species* in SBML terminology) to an edge, as in the case of inhibition processes; while Notes and Annotation are encoded in the set A, and may be associated with both nodes and edges.

Model construction and graphical representation were performed using the CellDesigner [52] software (version 4.4.2), which exploits the SBML coupled with SBGN notations to enable a detailed illustration of a pathway components as well as a flexible representation of complex biochemical interactions. To guarantee maximum informativeness when representing molecular complexes, we distinguish two classes of complexes: the first one corresponds to a biological complex (e.g., a protein complex) and the second one consists of a group of molecules which participate in a shared biological function (e.g., DNA damage, oxidative stress), herein referred to as a functional complex. To differentiate them we adopt the Reactome notation of “*Defined* and *Open* Sets, respectively (see Supplementary Materials for details). To avoid possible ambiguity in notation and to be compliant with the SBML terminology, we will refer to all interacting actors in the map (the nodes) as species, which include molecules, biological complexes, and functional complexes.

### 2.2. Overlap in Cellular Signaling

As mentioned above, the availability of physical stimuli transduction pathways is very scarce. To the best of our knowledge, only mechanotransduction has been provided in SBML format, with a recent update by our group [17]. Since transduction of physical stimuli is known to share anti-inflammatory activity [1], we exploited the machine-readable SBML representation to precisely search for such overlap, if any.

We used two different approaches to investigate these points of contact. The first, most intuitive approach, to which we refer as species-level overlap, searches directly for overlapping nodes between the two pathways (Section 2.2.1). The second approach, more sophisticated, searches for shared connected nodes and edges between the two; this pathway-level overlap is detailed in Section 2.2.2.

In both approaches, the search relies on identical MIRIAM IDs; however, biological and functional complexes are associated with more than one MIRIAM ID. In these case, two species from different maps are considered *equivalent* if they share at least one MIRIAM ID. Details on this procedure can be found in the Supplementary Materials.

#### 2.2.1. Species-Level Overlap

The first approach identifies all the equivalent species in the two maps. To achieve this, we used the *tidysbml* R package (version 1.6.0) [53], that extracts the list of all involved species and the related annotations from the SBML file. A simple overlap among the lists of species from the two maps identifies the shared molecules.

#### 2.2.2. Pathway-Level Overlap

The second approach uses the dedicated MIMO software (version 1.0) [54] to identify more subtle overlaps, by collecting the set of pathways, i.e., sets of nodes and connected edges that involve equivalent species in both maps. MIMO allows users to define a list of constraints for the computation of the overlap. In particular, the equivalence between species can be limited a priori, with exclusion criteria, which in our case are defined as follows:

• In our map, optical stimuli are all identified by the same MIRIAM ID (SBO:0000405, perturbing agent), regardless of the specific characteristics (wavelength, energy, etc.). Therefore, equivalence between such species is systematically excluded a priori in all analyses.
• Small molecules, with ChEBI ID, may or may not be explicitly represented in a map, depending on the level of resolution adopted in the literature. This may affect the overlap analysis in an uncontrolled manner. For this reason, we perform two separate analyses, one labeled *small molecules included*, where small molecules are matched, and one labeled *small molecules excluded*, where the equivalence between small molecules is explicitly excluded a priori.

Additional criteria include information about the compartments to which each species belongs. In practice, the match between equivalent species is allowed only if there is also a match between their associated compartments, case-labeled as *compartments included*. Alternatively, compartment information can be ignored; we named this case compartments *excluded*.

Finally, the pathways from different maps are not required to be identical up to a user-defined pathway length L. L indicates the number of edges that separate the nodes (molecules) of interest. That is, two pathways are considered similar and included in the overlap if they connect equivalent species, regardless of the similarity between the intermediate species involved in up to L reactions along the path. This is relevant in the intersection between the opto- and mechano-transduction maps, as overlap can be estimated regardless of differences in the granularity of the representations introduced during the reconstruction from the literature.

Overall, two species from different maps are considered equivalent for the purpose of the overlap analysis performed with MIMO if they share at least one associated MIRIAM ID and if their equivalence has not been explicitly excluded a priori by the above criteria. In summary, taking into account all possibilities relevant to us, we performed four comparisons for each length L, shown in Section 3 and in Supplementary Materials (Section Pathway-level Overlap), including further details on the use of MIMO.

### 2.3. Diffusion Analysis

Diffusion analysis is a widely used network-based method to identify subsets of nodes that are close to each other within a network [16]. In a nutshell, the method propagates a signal starting from a set of input nodes through the connections of the network, producing a proximity ranking that is specific to the wiring that represents the system modeled by the network. This can be used to assess which nodes participate in the same transmission of information. In our case, we are interested in analyzing how optical stimuli propagate within the network, and which nodes are ultimately perturbed, since this translates into a proxy of the molecules affected by a given stimulus.

We perform diffusion analysis using both individual input nodes as directly extracted from the literature (identified by ID = SBO:0000405 and shown in Table S1 of the Supplementary Materials), as well as sets of these nodes grouped according to the electromagnetic spectrum standard division, namely Green light (input nodes: Green, 532 nm), Red light (input nodes: 628 nm, 633 nm, 632.8 nm, 635 nm, 650 nm, 660 nm, 670 nm), and Near-IR (input nodes: NIR, 780 nm, 800 nm, 805 nm, 810 nm, 830 nm, 1068 nm, 1072 nm). We excluded from the analysis stimuli expressed in terms of energy (J) and absorbed dose (mGy). The analysis is performed using the built-in diffusion function of Cytoscape (version 3.10.4) [55] using standard settings. For each input, we collect the species, along with a ranking quantifying how strongly the signal propagates, and use this pre-ranked list to perform GSEA [56] GO enrichment analysis. The enrichment analysis is carried out using the GSEApy library [57], which returns a set of biological processes associated with the provided species. From these, we retain only the statistically significant terms (q-value < 0.05).

## 3. Results and Discussion

### 3.1. Optotransduction Map

Overall, the map consists of seven compartments, 177 species, and 305 reactions. It is available in two SBML files, optotransduction-core_celldesigner.xml and optotransduction-core_sbml.xml, generated using CellDesigner included in the Supplementary Materials, in the high-resolution Figure S1 and publicly available in the BioModels repository under the identifier MODEL2604100001 (Available online: https://www.biomodels.org/MODEL2604100001), where future extensions and updates will also be made available. Additional details, including the description of the differences between the two files, are provided in the Supplementary Materials (Section Optotransduction Map).

### 3.2. Mechano and Opto—Transduction Shared Pathways

#### 3.2.1. Species-Level Overlap

Table 1 lists the species in the optotransduction and mechanotransduction maps for which there is a match between the associated sets of MIRIAM IDs, as defined in Methods. Matches involving small molecules, i.e., species whose MIRIAM ID is a ChEBI identifier, are excluded from Table 1, for the sake of readability. A comprehensive list of all matches is provided in Table S2 of the Supplementary Materials.

#### 3.2.2. Pathway-Level Overlap

The overlap analysis performed with MIMO provides different results depending on the constraints on compartments, small molecules and input length L. From a preliminary analysis, we observed that the maximum pathway length in both the optotransduction and mechanotransduction maps is L = 15. For this reason, we performed the overlap analysis with MIMO using pathway lengths L ranging from 1 to 15, as shown in Table 2.

**Figure 1:**
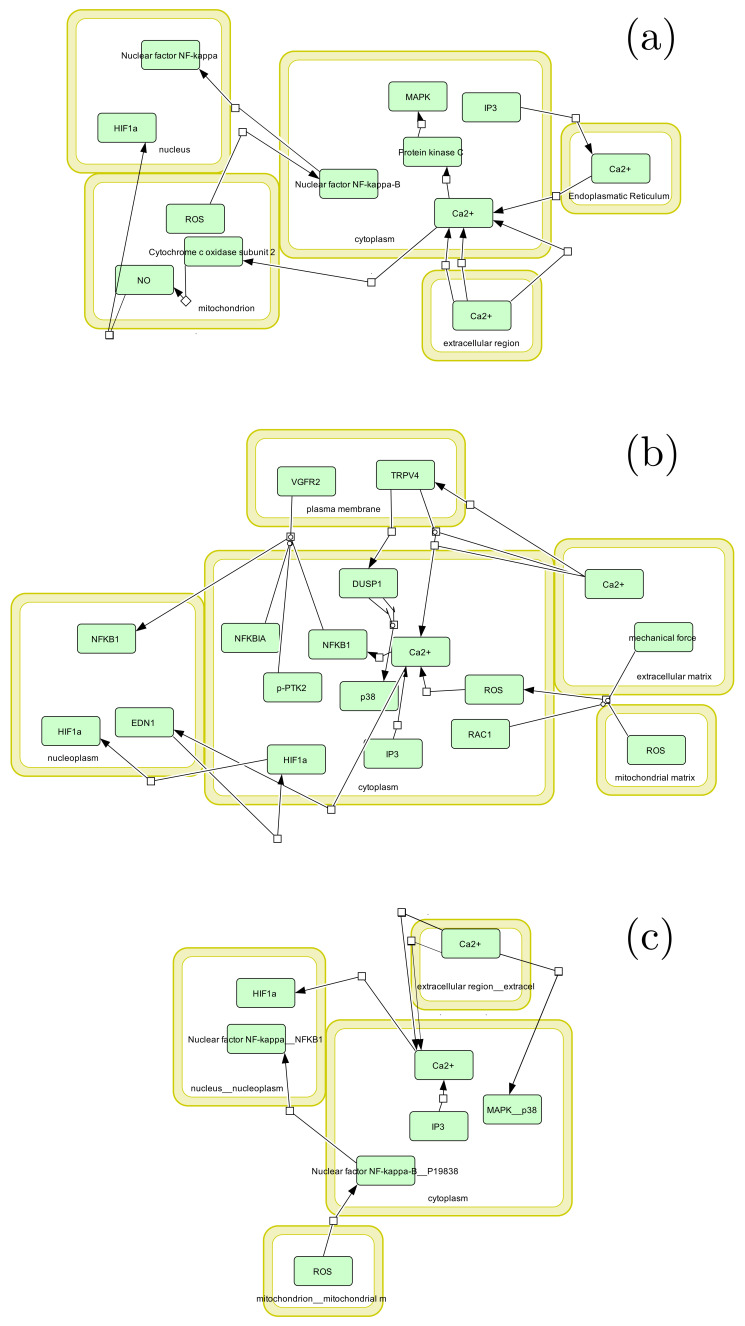
Pathway-level overlap between the optotransduction and mechanotransduction maps obtained with MIMO. (**a**) species from the optotransduction map involved in the overlap; (**b**) species from the mechanotransduction map involved in the overlap; (**c**) overlap between the two maps.

Overall, we found that the configuration providing the richest information, i.e., the most biologically interpretable results in terms of associated biological functions, is the one where both small molecules and compartments are considered, with input length L = 3, as shown in Figure 1. This value of L corresponds to considering as equivalent the pathways in the two maps that connect the same species through a number of reactions ranging from 1 to 3. We observe that the number of species involved in the overlap increases with L, from 2 species for L = 1 to 6 species for L = 3; increasing L beyond 3 does not increase the number of involved species (Figure S2 in the Supplementary Materials). Therefore, our analysis suggests that this level of granularity is the most appropriate for constructing the overlap between the optotransduction and mechanotransduction maps.

The overlap illustrated in Figure 1 shows that there is a common intracellular signaling machinery converging on calcium- and redox-dependent inflammatory mechanisms. Both types of stimuli converge towards cytosolic Ca^2+^ as a common second messenger, regulated by its extracellular influx and by inositol 1,4,5-trisphosphate (IP3)-mediated release from endoplasmic reticulum stores. This signaling pathway is closely related to the production of mitochondrial reactive oxygen species (ROS), thereby forming a bidirectional amplification loop for signal transduction. Downstream of these events, activation of stress kinases like p38 MAPK and transcription factors like NF-κB and HIF1α is observed, leading to the generation of inflammatory mechanisms. This shows that, in an inflammatory milieu, different types of physical stimuli—mechanical and optic—are integrated through a common signaling architecture involving Ca^2+^-ROS-MAPK- NF-κB- HIF1α, with the mitochondrion acting as an integrator. In particular, ROS and Ca^2+^ act as a second messenger that can be stimulated by both mechanical and optical stimuli; mitochondria are the core, as they produce ROS and NO, sense light through cytochrome oxidase and are sensitive to shear stress; p38MAPK and NF-κB are common pro-inflammatory actors, linking optical and mechanical stimuli to inflammation; HIF1α is stabilized by ROS and mechanical stress.

In addition to these predominantly pro-inflammatory aspects, specific components can mediate anti-inflammatory effects. In particular, in mechanotransduction, a low intensity stimulus, i.e., gentle mechanical strain, induces high expression of DUSP1 [58], which dephosphorylates p38 and JNK, limiting their inflammatory signaling with negative feedback that can be anti-inflammatory. The portion of the mechanotransduction map that incorporates this specific pathway is shown in Figure S3 of the Supplementary Materials, driven by stretch inputs. This signal follows the sequence stretch → TRPV4 → DUSP1. Consequently, DUSP1 acts as a direct regulator of p38 and JNK (MK08) signaling.

In optotransduction, a low intensity optical stimulus LLLT induces a low expression of NO and activates cytocrome c oxydase. Low/moderate NO can be anti-inflammatory, as it inhibits NF-κB and reduces adhesion molecules, while high NO is pro-inflammatory; mitochondrial cytochrome c oxidase, when activated by certain light wavelengths (i.e., PBM), increases ATP, reduces ROS, and can lower inflammation via mitochondrial retrograde signaling. In the optotransduction map, this connection is evident and similarly reported in Figure S3 of the Supplementary Materials. Specifically, red light and NIR stimuli, associated with low-level light therapy and PBM, are directly linked to cytochrome c oxidase subunit 2, which in turn is directly coupled to ROS and ATP production. These stimuli are also linked to NO through a signaling network involving Vascular Endothelial Growth Factor, oxidative stress enzymes, and cytochrome c oxidase subunit 2. Subsequently, NO connects to NF-κB via IKB and AP-1 signaling.

Thus, mechanotransduction anti-inflammatory effect is mainly linked to high DUSP1 expression, while optotransduction has two putative anti-inflammatory mechanisms, such as NO-dependent inhibition of NF-κB and light-induced mitochondrial modulation, that may counterbalance the pro-inflammatory signals. This result represents an important example of low-cost hypothesis generation. To the best of our knowledge, although the possibility to have shared biochemical mechanisms among different transductions has been vented [1,18], our approach enables the systematic search for such overlaps. In particular, it offers, for the first time, a rationale to compare gentle mechanical strain with NIR /red light stimuli. Indeed, it may be hypothesized that the Ca^2+^–ROS–MAPK–NF-κB–HIF1α pathway build a shared architecture of a cell-intrinsic detection system for bioenergetic and redox perturbations—what mechanical deformation and photon absorption ultimately produce inside the cell—with the mitochondrion being the central cellular integrator. Indeed, it directly absorbs red/NIR photons via cytochrome c oxidase, releasing NO and modulating ATP and ROS [59], while also responding to mechanical force transmitted through the cytoskeleton [60]. Mitochondrial ROS then activate NF-κB via IKK phosphorylation and stabilize HIF1α by inhibiting prolyl hydroxylases [61,62], while p38 MAPK—a kinase whose stress-reactive role stems from yeast osmo-sensing—is phosphorylated by both ROS and Ca^2+^-dependent cascade downstream to mechanical solicitation [63]. The anti-inflammatory divergence (DUSP1 in mechanotransduction vs. NO/COX modulation in optotransduction) operates downstream of this shared core, explaining why the two stimuli can be therapeutically interchangeable despite using different molecular resolution mechanisms. In brief, the cell does not recognize the physical origin of a perturbation at the level of its core stress-signaling network—only the upstream sensor and the downstream resolution effector differ.

Mechano- and opto-triggered paths converging to a similar anti-inflammatory activity could help with framing alternative anti-inflammatory therapies. For instance, patients hardly tolerating mechanical strain, or dermatological conditions impeding the usage of light, could take advantage of alternative stimuli. Experimental validation is clearly needed, but these results dramatically reduce the search space for comparable alternatives (i.e., gentle strain and NIR/red light, versus all combinations of mechanical stress and optical stimuli).

### 3.3. (Diffusion Analysis) Exploring Differential Biological Effects of Wavelengths

The diffusion analysis of the optotransduction network reveals both shared and distinct patterns (enriched GO biological processes) for a subset of input nodes, namely 405 nm (UVA), 532 nm (green light), 650 nm (red light), and 400–1100 nm (visible + near-IR). Table 3 shows the obtained results; full details can be found in Table S3 of SupplementaryTables.xlsx.

Other inputs, as well as aggregated stimuli (Green Light, Red Light, and Near-IR, introduced in Section 2.3), do not yield significant results. It may be surprising that specific wavelengths within a small range do not share the same type of enrichment. This is most likely due to the diverse objectives of the published studies, the experimental technology and the research question, often involving measurement limited to known/associated key molecules, while others may be neglected or overlooked. The way in which processes are explored across studies impact the resulting network topology and, in turn, the different levels of heterogeneity in the connections between input nodes and other species in the map, resulting in a broader or more restricted propagation of the diffusion signal, and, ultimately, in the results of the enrichment analysis.

With respect to the enrichment results, we observe that *Response to Peptide Hormone (GO:0043434)*, is enriched for all four stimuli. The lead genes, CRY1, SRC and SIRT1, are involved in circadian regulation and signaling pathways connected to light response. They could be considered as an integrative hub where CRY1, a core component of the circadian clock, links the external stimuli (400–1100 nm, 650 nm, and 405 nm) to the internal circadian clock, thus allowing its setting (entrainment). SRC is a non-receptor influencing several core processes through kinase-mediated signals. In the context of *Response to Peptide Hormone*, it could be a signal integrator/amplifier that, following the exposition to different wavelengths radiation, translates the light input from CRY1 and the metabolic input from SIRT1 into kinase-mediated signaling. In particular, SRC could potentiate the cellular response to a concurrent peptide hormone signal, thereby influencing its metabolic action in a light-entrained way.

In the following, enrichment results are discussed from a stimulus-centric perspective, to better highlight their similarities and differences. The analysis of these profiles will assume that negative regulation processes suggest suppressive or protective roles; cellular responses to TNF indicate inflammatory modulation; peptide hormone response suggests metabolic/endocrine effects; cell adhesion regulation impacts tissue organization.

**400–1100 nm (visible + near-IR).** The dominant effect of this broadband stimulus is negative regulation (biosynthesis and signaling) coupled with stress responses (TNF, hormone) and adhesion control. This suggests a protective, anti-inflammatory, and metabolically integrative role.

**650 nm (red light).** Red light predominantly negatively regulates gene expression and modulates TNF/hormone responses. Compared to 400–1100 nm, red light stimulus appears more focused on transcriptional control in a context of stress response.

**532 nm (green light).** Green light has the weakest overall enrichment (FDR values near 0.05). Its effect is a possible negative regulation of transcription (negative regulation of gene expression) and cellular component organization, suggesting a role in stabilizing cell architecture under stress (response to peptide hormone).

**UVA.** UVA most strongly activates peptide hormone response (FDR = 0.001) and also robustly regulates TNF signaling, gene expression, and cell organization. Its effects are broader and more potent than green light, though narrower than 400–1100 nm (which uniquely affects macromolecule biosynthesis and signal transduction regulation). The overall effect of UVA is a negative control of transcription (negative regulation of gene expression) and cellular architecture (negative regulation of cellular component organization) under conditions of cell stress (cellular response to TNF/response to peptide hormone).

Additional information on this analysis is provided in Supplementary Materials (Section Diffusion Analysis). These results represent a starting point as well as a word of caution when using light in clinical contexts: as we observed already for electrical stimuli in a simplified model of inflammation [64], the effect of physical triggers is context-dependent, in particular in inflamed versus physiological contexts, and in general under stress conditions. These results then recommend to explore the activation of these biological functions in differential stress contexts.

## 4. Conclusions

Our work offers several novelties of interest for the biomedical community concerned with the usage/effects of optical stimuli. First of all, we offer a carefully curated pathway of optotransduction; to the best of our knowledge, this is the first attempt of such breadth and certainly in the standard SBML format. Second, we explore the overlap of optotransduction with mechanotransduction, and identify a relevant mechanism associated with inflammation, whose activation can potentially be reconciled to optical or mechanical stimuli. The convergence of mechano- and opto-triggered paths to a similar anti-inflammatory activity offer a very interesting applicative perspective: this could help frame therapeutic alternatives, personalized not only to patients’ needs but also to clinical and cultural contexts. Finally, we explore the differential biological effects of different wavelengths, offering a first characterization with potential biomedical interest.

Limitations must also be highlighted. Clearly, the map is as precise as the literature we explored; this means that there is a potential for more extensive coverage of the topic, and that new research should be continuously included. Although this latter point represents good practice in computational biology, it should not be taken for granted. It must also be mentioned that, although rigorous double checking of the conversion from the literature to SBML has been done in several rounds and by all co-authors, the process still relies on manual curation, with its pros and cons. Also, inference from computational work comes with the advantage of lower experimental costs, and, of course, lower reliability. For this reason, the results we discuss must be recognized as hypotheses which can offer an advantageous starting point to design more expensive experimental work, based on solid rationale. The gold standard remains clinical validation, whose cost-effectiveness can take advantage from computational explorations.

## Figures and Tables

**Table 1:** Species-level overlap between the optotransduction and mechanotransduction maps based on MIRIAM IDs. * indicates that the IDs in parenthesis refer to the protein and its phosphorylated form, respectively.

Optotransduction Map		Mechanotransduction Map	
Name (Map ID)	MIRIAM ID	Name (Map ID)	MIRIAM ID
Nuclear factor NF-kappa-B (s22)	P19838_Q00653	S18 (s18)	P25963_P19838
		NFKB1 (s15; s16)	P19838
		p65/p50 dimer (s852)	Q04206_P19838
Dna-repair (s38)	O00206_Q99836	TLR 4 (s623)	O00206
Ox-stress enzymes (s63)	P00441_P04179_P04040_P09601_P35228_Q9Y5S8	SODM (s849)	P04179
AHR (s83)	P35869	AHR (s911)	P35869
		S917 (s917)	P27540_P35869
TNFA (s117)	P01375	TNFA (s937)	P01375
MAPK (s143)	P27361_P28482_Q16539_Q15759_P53778_O15264_P45983_P45984_P53779	MK08 (s570)	P45983
		* ERK1/2 (s957; s961)	P27361_P28482
		P38 (s956)	Q16539_O15264_Q15759_P5377
Nuclear factor NF-kappa-B (s170)	Q00653_P19838	S18 (s18)	P25963_P19838
		NFKB1 (s15; s16)	P19838
		p65/p50 dimer(s852)	Q04206_P19838
P2Y purinoceptor (s179)	Q9H244_Q9BPV8_Q15077_P47900_P51582_P41231_Q96G91_Q86VZ1	P2RY2 (s58)	P41231
		Complex1 (s65)	CHEBI%3A15422_P41231
JAK/STAT pathway (s253)	O60674_P42224_P40763_P42229	JAK2 (s872)	O60674
		STAT1 (s873)	P42224
		STAT3 (s874)	P40763
CYP1A1 (s272)	P04798	CYP1A1 (s920)	P04798
SRC (s273)	P12931	Complex3 (s842)	P12931_Q05397
		SRC (s912)	P12931
* CREB (s282; s284)	P16220_O43889_Q02930	CREB1 (s831)	P16220
HIF1a (s287)	Q16665	HIF1a (s561; s945)	Q16665
smad1-5-8 (s292)	Q15797_Q99717	* SM1/5 (s803; s804)	Q15797_Q99717_Q13485
p-38 (s297)	Q16539_O15264_Q15759_P53778	p38 (s956)	Q16539_O15264_Q15759_P5377
*CCND1* (s310)	595	*CCND1* (s193)	595
Smad (s345)	Q15796_P84022_Q13485	* SM2/3 (s844; s733)	Q15796_P84022_Q13485
		* SM1/5 (s803; s804)	Q15797_Q99717_Q13485
PI3K (s360)	P42336_O00329_P48736_Q8NEB9	PK3CG (s836)	P48736
SIR1 (s366)	Q96EB6	SIR1 (s826)	Q96EB6
CAMP-dependent protein kinase catalytic (s367)	P17612_P22694_P22612	KAPCB (s830)	P22694
SOX9 (s372)	P48436	SOX9 (s832)	P48436
		s924 (s924)	P48436_Q13950
IL1B (s380)	P01584	IL1B (s939)	P01584
IL8 (s384)	P10145	IL8 (s940)	P10145
IL6 (s385)	P05231	IL6 (s946)	P05231
TGF-beta (s390)	P01137_P61812_P10600	TGFB (s525)	P01137

**Table 2:** Summary of pathway-level overlap analysis. For each configuration of imposed constraints, the minimum input length that maximizes the number of species in the overlap is reported, along with the corresponding number of species. This number does not increase as L increases.

Small Molecules	Compartments	Minimum L	Overlap Size
Included	Included	3	6
Included	Excluded	7	10
Excluded	Included	1	2
Excluded	Excluded	7	4

**Table 3:** Results of the enrichment analysis sorted by statistical significance, performed from the diffusion analysis output. Term and ID columns: GO biological process. Lead Genes: genes contributing most to the enrichment score. Stimulus: name of the stimulus used in the diffusion. FDR q-val: q-value (adjusted False Discovery Rate) for each stimulus enrichment.

GO Term	GOID	Lead Genes	Stimulus	FDR q-Val
Negative Regulation of Macromolecule Biosynthetic Process	GO:0010558	SIRT1, CXCL8, TNF	400–1100 nm	0.023
Cellular Response to Tumor Necrosis Factor	GO:0071356	SIRT1, CXCL8, TNF, TP53	400–1100 nm 650 nm UVA	0.027 0.012 0.014
Regulation of Cell Adhesion	GO:0030155	SRC, CXCL8, TNF	400–1100 nm	0.031
**Response to Peptide Hormone**	**GO:0043434**	**CRY1, SRC, SIRT1**	**400–1100 nm** **532 nm** **650 nm** **UVA**	**0.039** **0.048** **0.029** **0.001**
Negative Regulation of Signal Transduction	GO:0009968	CRY1, CXCL8, TNF	400–1100 nm	0.047
Negative Regulation of Gene Expression	GO:0010629	HSPA1B, SIRT1, TNF, CXCL8	532 nm 650 nm UVA	0.044 0.005 0.026
Negative Regulation of Cellular Component Organization	GO:0051129	SRC, HSPA1B, TNF	532 nm UVA	0.049 0.032

In bold, the GO term in common to all stimuli.

## Data Availability

The data presented in this study are openly available in [https://biomodels.org/] [https://www.biomodels.org/search?query=MODEL2604100001] [MODEL2604100001].

## Supplementary Materials

The following supporting information can be downloaded at https://www.mdpi.com/article/10.3390/biom16060859/s1, File S1: Supplementary Figures and Tables. Table S1: List of species representing stimuli within the optotransduction map; Table S2: Full list of matches between species in Optotransduction and Mechanotransduction maps according to Equation (S1); Table S3: Details of the nature of the overlap identified by MIMO, with the surrounding environment shown in Figure S3; Table S4: Full results of the enrichment analysis performed from the diffusion output, as described in the main text; Figure S1: overlap results; Figure S2: number of different species in the overlap for the various lengths L; Figure S3: Cytoscape rendering of the sub-pathways representing the stimulus-to-overlap paths in the mechano; File S2: Optotransduction core celldesigner map.
