# Supplementary material for: Optotransduction Pathway, Exploring Connections with Inflammation
Source: Biomolecules. 2026 Jun 11;16(6):859. doi: 10.3390/biom16060859 (PMC13296732; doi:10.3390/biom16060859)
Supplement: Supplementary file 1 [file biomolecules-16-00859-s001.zip › File S1.pdf]

Supplementary Materials

Optotransduction pathway, exploring connections with inflammation

Alessandro Ravoni[1§], Veronica Paparozzi[1§], Tiziana Guarnieri[1,2§], Cecilia Sanzini[1,3], Luigi Manni[3], Christine Nardini[1*]

[1] Consiglio Nazionale delle Ricerche, Istituto per le Applicazioni del Calcolo "Mauro Picone", 00185 Roma, Italy alessandroravoni@cnr.it, veronicapaparozzi@cnr.it, christine.nardini@cnr.it
[2] Dipartimento di Scienze Biologiche, Geologiche e Ambientali (BIGEA), University of Bologna, 40100 Bologna, Italy tiziana.guarnieri@unibo.it
[3] Centro Interdipartimentale di Ricerca Industriale - CIRI Scienze della Vita e Tecnologie per la Salute, Università di Bologna, 40126 Bologna, Italy

[4] Consiglio Nazionale delle Ricerche, Istituto di Farmacologia Traslazionale, 00185 Roma, Italy, ceciliasanzini@gmail.com, luigi.manni@ift.cnr.it

Optotransduction Map

The primary objective of the map is to provide a comprehensive 'in silico' representation of the downstream mechanisms elicited by electromagnetic signals, specifically focusing on interactions with inflammatory mechanisms.

We first identified the principal acceptors involved in PBM, including cytochrome c oxidase (CCO), opsins, cryptochromes, and photosensitive ion channels. We subsequently examined how different stimuli, i.e. electromagnetic radiation characterized either by different wavelengths or by absorbed energy (see Table S1), modulate these receptors, and the mechanisms by which their activation initiates signal transduction pathways that promote anti-inflammatory responses. Among the primary pathways identified is the activation of several growth factors, including transforming growth factor beta (TGF-β), vascular endothelial growth factor (VEGF), and hepatocyte growth factor (HGF). Other triggered events are apoptotic processes mediated by members of the Bcl-2 family, whose activity is regulated through c-Jun N-terminal kinase (JNK) phosphorylation. LLLT is reported to modulate the activity of key transcription factors, including nuclear factor kappa B (NF-κB), hypoxia-inducible factor (HIF), and activator protein 1 (AP-1), thereby contributing to the regulation of inflammatory and cellular stress responses.

Stimuli

The stimuli considered comprise both discrete (single wavelength) emissions and defined wavelength ranges of electromagnetic radiation, encompassing (near-)infrared, visible light, and ultraviolet radiation (see Table S1 for a complete list). Moreover, there are stimuli expressed in milligray (mGy) and joule per kilogram (J) units, as well as ultraviolet radiation in presence of psoralen, a photosensitizing drug (PUVA).

Electromagnetic stimuli
UV (100-400)

400-1100 nm
405 nm
532 nm
628 nm
632.8 nm
633 nm
635 nm
650 nm
660 nm
670 nm
780 nm
800 nm
805 nm
810 nm
830 nm
1068 nm
1072 nm
NIR
PUVA
green
blue
light-dark cycle
20-50 mGy
LDIR (<= 200 mGy)
< 100 mGy
100 mGy
10-50 J
30 J

Table S1. List of species representing stimuli within the optotransduction map.

Implementation

Through the CellDesigner software, two XML-formatted files were generated to represent the map: a native CellDesigner file (optotransduction-core_celldesigner.xml), which exploits SBGN for graphical representation, and a corresponding pure-SBML version, which was exported in SBML Level 2 Version 4 format (optotransduction-core_sbml.xml). Both files consist of Compartment, Species and Reaction objects. As already stated in the main document, all three object types are characterized by both specific attributes and information, which are included in Notes and Annotation tags. Notably, supplementary human-readable details were provided using the Notes element, whereas structured metadata were encoded using the Annotation element. The former was used to provide textual information regarding the different nature of molecular complexes (as defined in Section 2.1 of the main document), specifically distinguishing functional complexes by indicating whether the grouped species represent alternative components in a functional complex (a "DefinedSet") or constitute a set whose members cannot be explicitly enumerated (an "OpenSet"), following Reactome specifications. The latter, instead, incorporates MIRIAM-compliant identifiers to ensure unambiguous cross-referencing by mapping species components to their exact biological counterparts (via the "bqbiol:is"

and “bqbiol:hasPart” qualifiers) and by linking associated bibliographic references to PubMed records (via the “bqbiol:isDescribedBy” tag). In accordance with SBML specifications, species associated either with multiple distinct identifiers or with repeated instances of the same identifier (i.e. complexes) were annotated using the ‘bqbiol:hasPart’ tag, whereas species related to a single, unique identifier were annotated using the ‘bqbiol:is’ tag. In addition to standard SBML tags, the CellDesigner file includes further information regarding species classification, allowing the specification of whether a species is defined as a “protein,” “gene,” “complex,” “simple molecule,” or “unknown”. Stimuli were categorized under the “unknown” class, in the absence of better labels. Moreover, the file includes tags (e.g., CompartmentAlias, SpeciesAlias) to equip SBML with a graphical model, in which compartments are represented as boxes containing species; species are nodes displayed using different visualizations depending on their specific function (e.g., protein, receptor, complex, gene, ion, ion channel); and reactions are edges connecting either multiple species or a species and another reaction (as in events such as catalysis or inhibition). The reactions mainly used in the map were transitional, translocation, and inhibition reactions (https://www.celldesigner.org/documents/CellDesigner4ExtensionTagSpecificationE.pdf). To guarantee homogeneity and interoperability, including overlap analysis with MIMO with respect to the exclusion or inclusion of compartments, compartment names were made consistent with those in the mechanotransduction map.

Species-level overlap

Optotransduction map		Mechanotransduction map	
**Name (map id)**	**MIRIAM ID**	**Name (map id)**	**MIRIAM ID**
ROS (s12)	CHEBI%3A26523	ROS (s850)	CHEBI%3A26523
		ROS (s851)	
Nuclear factor NF-kappa-B (s22)	P19838_Q00653	S18 (s18)	P25963_P19838
		NFKB1 (s15)	P19838
		NFKB1 (s16)	
		p65/p50 dimer (s852)	Q04206_P19838
Dna-repair (s38)	O00206_Q99836	TLR 4 (s623)	O00206
Ox-stress enzymes (s63)	P00441_P04179_P04040_P09601_P35228_Q9Y5S8	SODM (s849)	P04179
AHR (s83)	P35869	AHR (s911)	P35869

		S917 (s917)	P27540_P35869
TNFA (s117)	P01375	TNFA (s937)	P01375
Ca2+ (s139)<br>Ca2+ (s169)<br>Ca2+ (s261)	CHEBI%3A29108	Ca2+ (s423)<br>Ca2+ (s425)	CHEBI%3A29108
MAPK (s143)	P27361_P28482_Q16539_Q15759_P53778_O15264_P45983_P45984_P53779	MK08 (s570)	P45983
		ERK1/2 (s957)<br>p-ERK1/2 (s961)	P27361_P28482
		P38 (s956)	Q16539_O15264_Q15759_P5377
NO (s157)<br>NO (s332)	CHEBI%3A16480	NO (s829)	CHEBI%3A16480
ATP (s158)<br>ATP (s178)	CHEBI%3A15422	ATP (s57)<br>ATP (s78)	CHEBI%3A15422
		Complex1 (s65)	CHEBI%3A15422_P41231
Nuclear factor NF-kappa-B (s170)	Q00653_P19838	S18 (s18)	P25963_P19838
		NFKB1 (s15)<br>NFKB1 (s16)	P19838
		p65/p50 dimer (s852)	Q04206_P19838
P2Y purinoceptor (s179)	Q9H244_Q9BPV8_Q15077_P47900_P51582_P41231_Q96G91_Q86VZ1	P2RY2 (s58)	P41231
		Complex1 (s65)	CHEBI%3A15422_P41231
JAK/STAT pathway (s253)	O60674_P42224_P40763_P42229	JAK2 (s872)	O60674
		STAT1 (s873)	P42224

		STAT3 (s874)	P40763
CYP1A1 (s272)	P04798	CYP1A1 (s920)	P04798
SRC (s273)	P12931	Complex3 (s842)	P12931_Q05397
		SRC (s912)	P12931
CREB (s282) p-CREB (s284)	P16220_O43889_Q02930	CREB1 (s831)	P16220
HIF1a (s287)	Q16665	HIF1a (s561) HIF1a (s945)	Q16665
smad1-5-8 (s292)	Q15797_Q99717	SM1/5 (s803) p-SM1/5 (s804)	Q15797_Q99717_Q13485
p-38 (s297)	Q16539_O15264_Q15759_P53778	p38 (s956)	Q16539_O15264_Q15759_P5377
CCND1 (s310)	595	CCND1 (s193)	595
Smad (s345)	Q15796_P84022_Q13485	p-SM2/3 (s733) SM2/3 (s844)	Q15796_P84022_Q13485
		SM1/5 (s803) p-SM1/5 (s804)	Q15797_Q99717_Q13485
PI3K (s360)	P42336_O00329_P48736_Q8NEB9	PK3CG (s836)	P48736
SIR1 (s366)	Q96EB6	SIR1 (s826)	Q96EB6
CAMP-dependent protein kinase catalytic (s367)	P17612_P22694_P22612	KAPCB (s830)	P22694
SOX9 (s372)	P48436	SOX9 (s832)	P48436

		s924 (s924)	P48436_Q13950
PGE2 (s378)	CHEBI%3A15551	PGE2 (s828)	CHEBI%3A15551
IL1B (s380)	P01584	IL1B (s939)	P01584
IL8 (s384)	P10145	IL8 (s940)	P10145
IL6 (s385)	P05231	IL6 (s946)	P05231
TGF-beta (s390)	P01137_P61812_P10600	TGFB (s525)	P01137
IP3 (s362)	CHEBI%3A203600	IP3 (s424)	CHEBI%3A203600

Table S2. Full list of matches between species in Optotransduction and Mechanotransduction maps according to Equation (S1) in the main document.

Pathway-level Overlap

Because MIMO operates based on species names, identifying similarities between two maps only when these names are identical, the intersection of processes involving isoforms or complexes that are not identically named in the two maps but share common molecular components may be missed.
For this reason, additional information is provided to the software before the overlap analysis, specifically instructing MIMO to consider certain species as equivalent based on predefined constraints. Note that this procedure is an option provided by MIMO itself, which allows the user to supply an input file specifying a set of species to be treated as equivalent (or distinct) regardless of their names.
In particular, we rely on MIRIAM ID as an attribute to determine the equivalence between two species. For individual molecules, this typically corresponds to a unique identifier characterizing the molecule. However, a single complex can be associated with multiple MIRIAM ID, each describing one of its molecular components. Similarly, different MIRIAM ID may be associated with different isoforms of the same species. This introduces the constraint formalized in Equation (S1) of the main text:

(S1) $n1 = n2 \Leftrightarrow M(n1) \cap M(n2) \neq \emptyset$,

where M(n) denotes the set of MIRIAM ID associated with species n. For example, consider a hypothetical complex C1 from map I associated with the IDs IDa and IDb, and two complexes, C2 and C3, from map II associated with the IDs IDa and IDc, and IDd and IDe, respectively. Complexes C1 and C2 would therefore be considered equivalent, as they share the ID IDa.

Once an equivalence relationship is established, MIMO searches for pathways in both maps that connect species identified as equivalent, in order to build the overlap. For example, if there are pathways $P_A = \{r1 \rightarrow r_2 \rightarrow r_3 \rightarrow \ldots\}_A$ and $P_B = \{r1 \rightarrow r_2 \rightarrow r_3 \rightarrow \ldots\}_B$ connecting species n_1 to species n_2 in maps A and B respectively, the connection from n_1 to n_2 is added to the overlap.
The pathways P_A and P_B are considered similar and included in the overlap if they connect the equivalent species (n_1 and n_2), regardless of the similarity between the intermediate species involved in up to L reactions along the path from n_1 to n_2, where L is a user-defined parameter.

MIMO also allows either to include the information about compartments, considering as identical only the equivalent species belonging to the same compartments in the two maps, or to ignore the spatial component of the compartments.

Finally, it is possible to provide the software with a list of forbidden equivalence relationships between species in the form $n1 \neq n2$. This additional input takes precedence over all other constraints; that is, two species n1 and n2 declared as non-equivalent in the input will be considered non-equivalent by MIMO, even if their MIRIAM IDs satisfy Equation (S1).

Results

The following Figure S1 shows the overlaps obtained with MIMO for different configurations (including or excluding small molecules and compartments, see Section 2.2 in the main text for further details), considering the minimum length that, in each configuration, maximizes the number of species in the overlap.
We also summarize in Figure S2 the results obtained for the different configurations and the various lengths L.

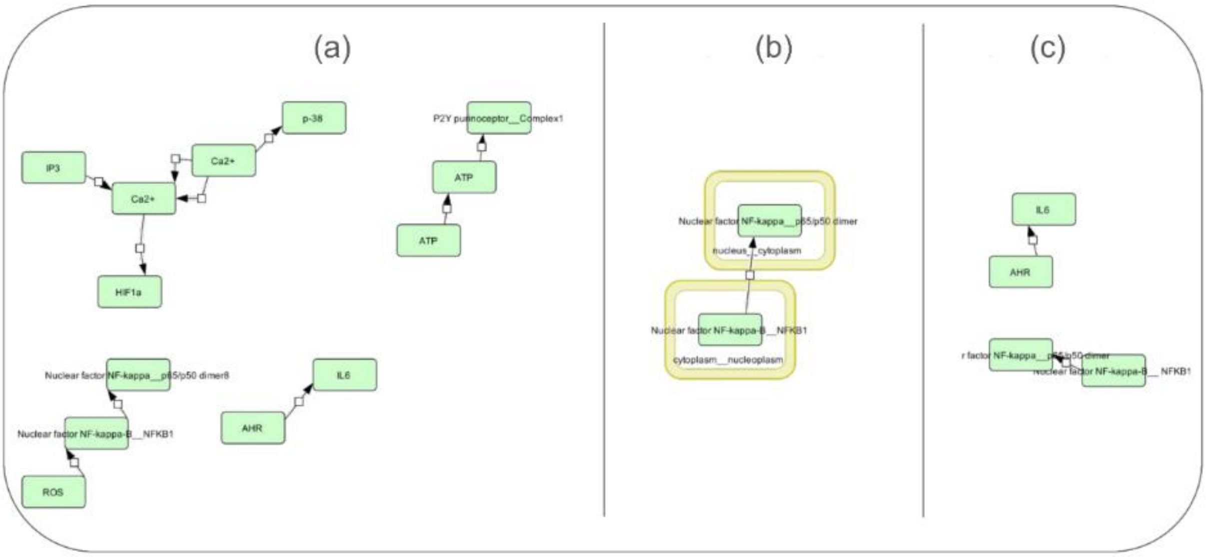

Figure S1: overlap results. Panel (a): small molecules included in the overlap analysis, compartments excluded. Panel (b): small molecules excluded, compartments included. Panel (c): small molecules and compartments excluded. Each panel shows the pathways obtained for the minimum length that, in each configuration, maximizes the number of species.

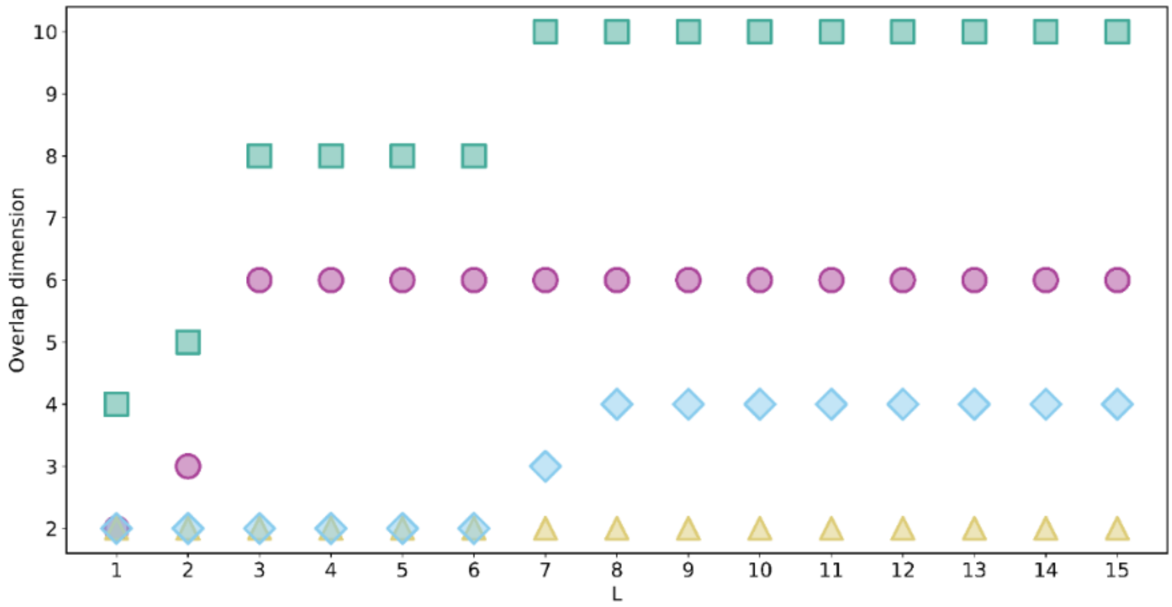

Figure S2: number of different species in the overlap for the various lengths L. Magenta circles: small molecules and compartments included in the overlap analysis. Green squares: small molecules included, compartments excluded. Yellow triangles: small molecules excluded, compartments included. Cyan diamonds: small molecules and compartments excluded.

Below, we provide a more in-depth discussion of the pathways in the mechanotransduction and optotransduction maps that involve the species appearing in the overlap obtained when including both small species and compartments, for L=3, as discussed in the main text.

Mechanotransduction Pathway: In this pathway, physical forces are converted into biochemical signals. Notably, these events happen in an inflammatory milieu. Here, we propose a mechanotransduction cascade initiated by an upstream mechanical input acting on the extracellular matrix within an inflammatory milieu. The mechanical cue activates the membrane receptors TRPV4 and VGFR2 (Vascular endothelial growth factor receptor 2, also known as KDR or Flk-1). TRPV 4 is a mechanosensitive ion channel mediating Ca2+and other cations influx, while VGFR2 has a kinase activity and in this case, in the absence of its ligand VEGFA, is activated through a mechanically induced phosphorylation. From their activation, two convergent early signaling events originate: the influx of extracellular Ca^{2+} into the cytoplasm and the production of IP_3, which stimulates the release of Ca2+ from internal stores. These small molecules act as key second messenger regulating ion signaling and downstream kinases, including the p38 MAPK, which is controlled by negative feedback by DUSP. Ca2+vinflux ignites ROS production through the activation of RAC, a GTPase involved in cytoskeleton dynamics. NF-kB (p65/p50 dimer) is activated in the cytoplasm by the IKK complex and is stabilized by IκBα until phosphorylation releases it. Another nuclear output is HIF1α, a transcription factor induced by hypoxia, a phenomenon usually occurring at the edges of wounds. HIF1 α promotes wound healing through the activation of angiogenic factors and stimulating the migration of fibroblasts and keratinocytes (not shown). HIF1α is stabilized by ROS and, when coupled to NF-kB, promotes cytokines expression and metabolic shifts. After its translocation to the nucleus, HIF1α activates the transcription of the gene EDN1, which contributes to cell migration. EDN1, with a feedback memechanism, can stabilize HIF1α.

Optotransduction Pathway: this pathway involves light-sensitive processes that converge on inflammatory processes. In the present model, optotransduction integrates photic stimuli into inflammatory pathways through a redox- and mitochondria-centered signaling cascade. As illustrated

in page 2 of the MIMO analysis, the pathway starts with photoreceptors or chromophore activation (not shown), that mediate the influx of Ca2+ from the extracellular space to the cytoplasm. As in the mechanotransduction pathway, this endows the activation of IP3 , which stimulates the release of endoplasmic Ca2+. The increase in cytosolic Ca^{2+} contributes to the activation of stress-responsive kinases such as p38 MAPK, as part of an inflammatory signal, and drives the activation of the mitochondrial cytochrome c oxidase, leading to the generation of reactive oxygen species (ROS) and nitric oxide (NO). These redox mediators operate as primary intracellular signals, promoting the activation of downstream pathways, including NF-κB and HIF1α. Overall, this pathway highlights the convergence of mitochondrial redox activity and calcium-dependent signaling in mediating optically induced inflammatory responses.

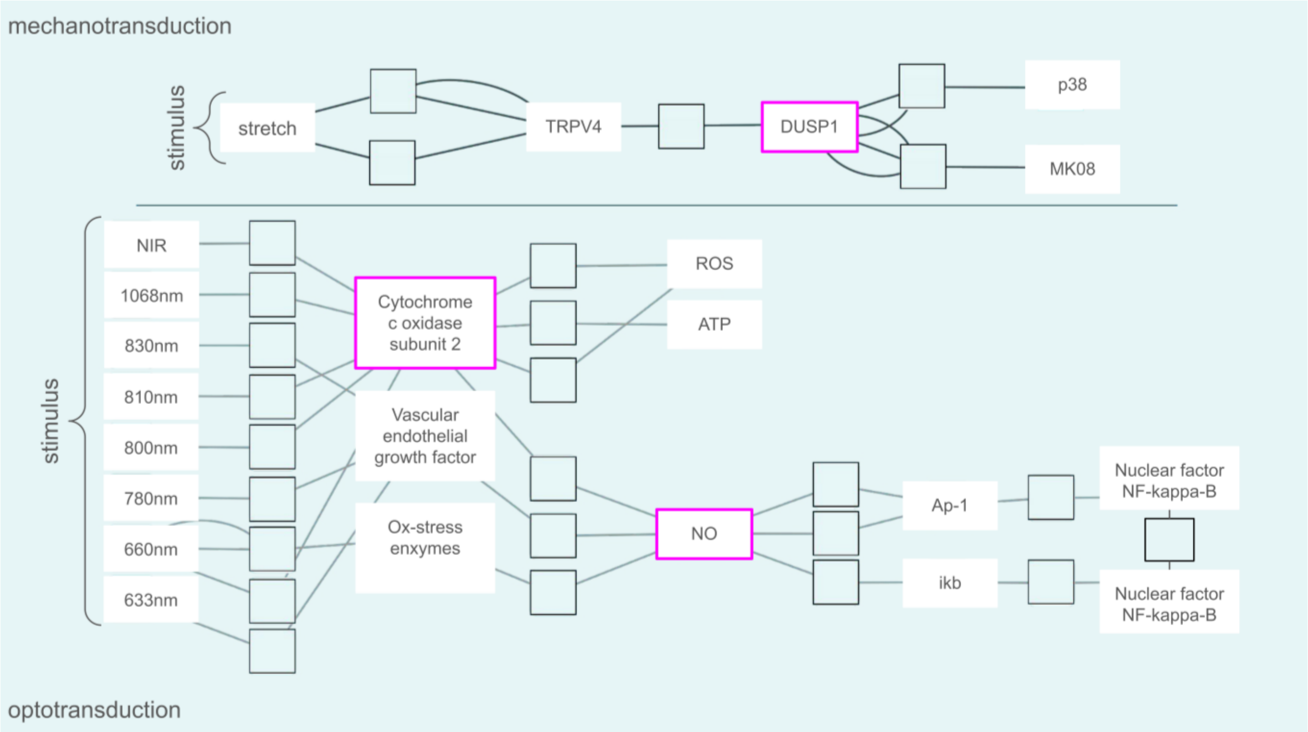

Figure S3 Cytoscape rendering of the sub-pathways representing the stimulus-to-overlap paths in the mechano (top) and opto-transduction maps (bottom). White squares represent the species, light blue squares represent the reactions. Key species for anti-inflammatory processes are highlighted with magenta outlines. These images present in more detail the non-overlapping, stimulus-specific chain of events that lead to the common mechanism highlighted by MIMO and discussed in the main paper. Details on the species involved are provided in Table S3.

**FEATURE**	**MECHANOTRANSDUCTION**	**OPTOTRANSDUCTION**	**OVERLAP**
**Stimulus**	Mechanical force	Light	
**Primary sensors**	TRPV4, VGFR2	Cytochrome c oxidase	
**Early mediators**	ROS, Ca^{2+} influx	ROS, Ca^{2+}, NO	ROS, Ca^{2+}
**Kinase pathways**	MAPK/p38	MAPK/p38	p38 MAPK
**Transcription factors**	NF-κB, HIF1α	NF-κB, HIF1α	NF-κB, HIF1α
**Key organelles**	Plasma membrane, ER, mitochondrion	Mitochondrion, ER	Mitochondrion, ER

**Inflammatory context**	NF-κB, HIF1α	NF-κB, HIF1α	NF-κB, HIF1α
**Anti-inflammatory context**	DUSP1	NO, Cytochrome c oxidase	

Table S3 Details of the nature of the overlap identified by MIMO, with the surrounding environment shown in Figure S3.

Diffusion Analysis

Stimulus	Term	Lead genes	Tag %	Gene %	FDR q-val
400-1100 nm	GO:0010558	SIRT1, CXCL8, TNF	3/3	13.11	0.023
400-1100 nm	GO:0071356	SIRT1, CXCL8, TNF, TP53	4/4	22.95	0.027
400-1100 nm	GO:0030155	SRC, CXCL8, TNF	3/3	13.11	0.031
400-1100 nm	GO:0043434	CRY1, SRC, SIRT1	3/3	8.2	0.039
400-1100 nm	GO:0009968	CRY1, CXCL8, TNF	3/3	13.11	0.047
532 nm	GO:0010629	HSPA1B, SIRT1, TNF, CXCL8	4/4	19.67	0.044
532 nm	GO:0043434	SRC, CRY1, SIRT1	3/3	9.84	0.048
532 nm	GO:0051129	SRC, HSPA1B, TNF	3/3	11.48	0.049
650 nm	GO:0010629	HSPA1B, SIRT1, TNF, CXCL8	4/4	16.39	0.005
650 nm	GO:0071356	SIRT1, TNF, CXCL8, TP53	4/4	19.67	0.012
650 nm	GO:0043434	CRY1, SRC, SIRT1	3/3	9.84	0.029
UVA	GO:0043434	CRY1, SRC, SIRT1	3/3	8.2	0.001
UVA	GO:0071356	SIRT1, TNF, CXCL8, TP53	4/4	19.67	0.014
UVA	GO:0010629	SIRT1, TNF, HSPA1B, CXCL8	4/4	16.39	0.026
UVA	GO:0051129	SRC, TNF, HSPA1B	3/3	11.48	0.032

Table S3. Full results of the enrichment analysis performed from the diffusion output, as described in the main text. Stimulus column: name of the stimulus used in the diffusion. Term column: identified

GO process. Lead Genes: leading edge genes. Tag %: percentage of the gene set before the running enrichment peak. Gene %: percentage of the gene list before the running enrichment peak. FDR q-val: q-value (adjusted False Discovery Rate). Only statistically significant results (FDR q-val < 0.05) are reported.

In addition to the **GO:0043434** Term common to all the four stimuli and discussed in the main text, every stimulus involves also other functions that have the potential to make its effect unique, or at least better targeted to specific biological activities.

Negative Regulation of Gene Expression (GO:0010629): this process is significant for *650 nm (red), 532 nm (green), and 405 nm (UVA) stimuli*. Considering the co-occurrence of HSPA1B, SIRT1, TNF, CXCL8 lead genes, it could be hypothesized by a coupled stress response/inflammatory feedback pathway. Here, the exposition to the above-mentioned wavelengths ignites the generation of Reactive Oxygen Species. This leads to the activation/increase of TNF signaling, which turns on CXCL8 (Interleukin-8) expression, probably via NF-κB activation. Interestingly, SIRT-1 is a well-known negative regulator of NF-κB-related transcription inflammatory and stress-response pathways. So, its activation fits well with the negative feedback part of this circuit where HSPA1B (HSP70) could exert a putative protective role as chaperone for misfolded proteins originated by cell stress and a stabilizer for IκB (the inhibitor of NF-κB), thus supporting the negative regulation of NF-κB and the attenuation of the inflammatory signaling.

Cellular Response to Tumour Necrosis Factor (GO:0071356): this pathway recurs in all stimuli that share the key genes — SIRT1, TNF, CXCL8, and TP53 — except for 532 nm radiation, thus suggesting that they play a central role in the response to light stress, likely by influencing inflammation (see above for a full explanation about SIRT1, TNF, CXCL8) and cell death pathways (TP53). We note that this process is additonally associated for 532 nm radiation when considering a FWER p-value < 0.05, as shown in Table S3.

Negative Regulation of Macromolecule Biosynthetic Process (GO:0010558) and **Regulation of Cell Adhesion (GO:0030155)** are uniquely enriched for *400–1100 nm stimulus*. Notably, they share the same gene set (CXCL8, and TNF), whose coordinated action likely reflects an inflammatory, self-limited (SIRT1) light-induced stress response. This limits energetically costly anabolic processes and remodels cell adhesion to either reinforce cellular positioning or permit migration. This coordinated strategy is orchestrated by SRC, a master signaling kinase that regulates both adhesion proteins and transcription factors/signaling nodes involved in biosynthetic pathways (i.e.: MAPK/STAT). TNF and CXCL8, as described above, are not only potent inflammatory cytokines, as TNF also represses protein translation and modulates integrin expression and activity, whereas CXCL8 promotes cell migration, a process that requires tightly controlled, SRC-mediated adhesion turnover.

Interestingly, **400–1100** nm enriches also for **Negative Regulation of Signal Transduction (GO:0009968)**, whose master gene set includes CRY1, CXCL8, and TNF and excludes SRC, indicating a widespread damping of signaling networks strictly linked to circadian rhythm (CRY1).

Negative Regulation of Cellular Component Organization (GO:0051129), is uniquely enriched for 532 nm and 405 nm stimuli. The gene dataset involves SRC, HSPA1B, and TNF, probably triggering a protective cellular "freeze" response. This strategy, generated from 532 nm and 405 nm exposition, could be a response to photochemical stress finalized at maintaining structural integrity through the modulation of cell cytoskeleton or structural reorganization. Unlike the pathways described above, the

absence of CXCL8 in the relevant dataset favors the remodeling and static consolidation of the cellular structure. From this, it is possible to hypothesize that, unlike the other wavelengths, 532 nm and 405 nm stimuli prioritize structural stability and damage containment over plasticity.
