# Supplementary figures and images for: Optotransduction Pathway, Exploring Connections with Inflammation
Source: Biomolecules. 2026 Jun 11;16(6):859. doi: 10.3390/biom16060859 (PMC13296732; doi:10.3390/biom16060859)

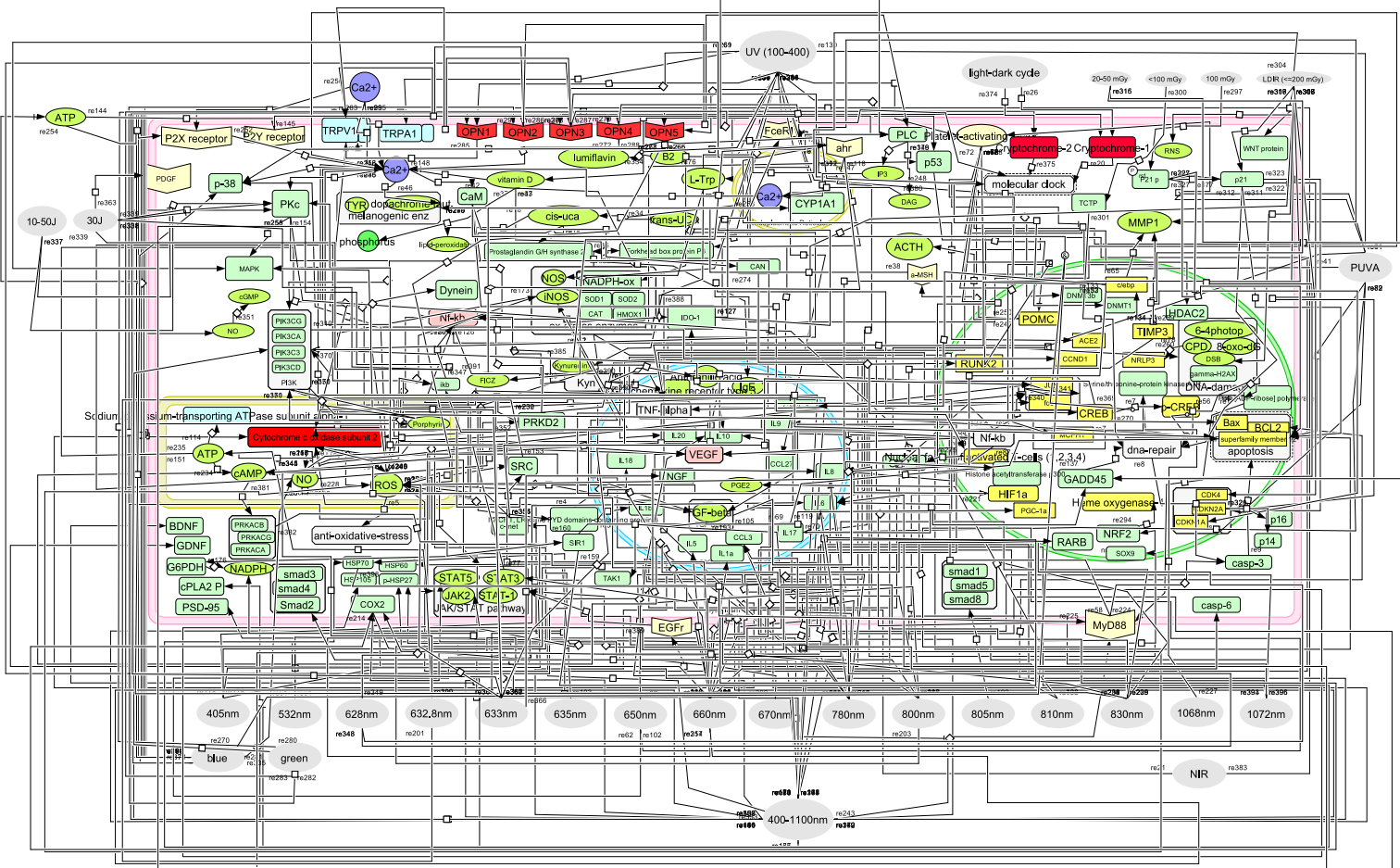

Supplement: Supplementary file 1 [file biomolecules-16-00859-s001.zip › File S2.pdf]
